# Interventional anti-reflux management for gastro-oesophageal reflux disease in lung transplant recipients: a systematic review and meta-analysis

**DOI:** 10.1007/s00464-024-11392-8

**Published:** 2024-11-25

**Authors:** Oliver Krahelski, Iihan Ali, Christopher Namgoong, Kavita Dave, Anna Reed, Hutan Ashrafian, Marcus Reddy, Omar Khan, Bibek Das, Matyas Fehervari

**Affiliations:** 1 Ashford and St Peters Hospital NHS Foundation Trust, Chertsey, UK; 2 Imperial College London, Hammersmith Hospital Campus, Du Cane Road, London, UK; 3 Royal Brompton and Harefield Hospitals, Part of Guy’s and St Thomas’ NHS Foundation Trust, London, UK; 4 Department of Bariatric Surgery, St George’s Hospital, London, UK; 5 Population Sciences Department, St George’s University of London, London, UK; 6 Department of Upper Gastrointestinal Surgery, Maidstone and Tunbridge Wells NHS Trust, Tunbridge Wells, UK

**Keywords:** Lung transplant, Anti-reflux surgery, Fundoplication, Radiofrequency ablation, Magnetic sphincter augmentation

## Abstract

**Introduction:**

Gastroesophageal reflux disease (GORD) and aspiration are risk factors in the development of bronchiolitis obliterans syndrome (BOS) in the lung transplant population. The aim of this study was to investigate if allograft function and survival improved after anti-reflux surgery (ARS) in lung transplant recipients.

**Methods:**

In accordance with PRISMA guidelines, we conducted a systematic search of MEDLINE, Embase, and the Cochrane library databases from inception until 13/01/2024. Articles reporting outcomes of ARS following lung transplantation were included. A random effects model was used for meta-analysis.

**Results:**

The search identified 20 which were used for quantitative analysis. Overall, FEV1 and rate of change of FEV1 had improved following ARS by 0.141 L/s (95% CI; −02.82, −0.001) and −1.153 mL/d (95% CI; −12.117, −0.188), respectively. Survival hazard ratio post-ARS was 0.39 (95% CI; 0.19, 0.60). Nissen fundoplication was the most effective anti-reflux procedure with the greatest effect on reduction in the rate of change of FEV1, with an improvement of −2.353 mL/d (95% CI; −3.058, −1.649).

**Conclusion:**

ARS in lung transplant recipients improves allograft function and survival. Given the increased incidence of GORD in lung transplant recipients, there should be a low threshold for investigation of GORD and subsequent ARS.

**Supplementary Information:**

The online version contains supplementary material available at 10.1007/s00464-024-11392-8.

Lung transplantation remains the final treatment option for patients suffering from end-stage pulmonary diseases, prolonging life and preventing respiratory failure. Despite modern surgical techniques and immunosuppressive regiments, there remain a myriad of complications. BOS is widely recognised as the disease which poses the biggest threat to long-term allograft function.

The aetiology is understood to include a fibroinflammatory process with multiple triggers. Along with acute cellular rejection, infection, medication, and ischaemia–reperfusion injury, GORD is profoundly involved in the pathological processes resulting in BOS [1, 2]. Reduced airway sensation following lung transplant dampens the cough reflex impairing mucociliary clearance resulting in microaspiration and subsequent allograft dysfunction [3]. Moreover, GORD disproportionately affects lung transplant recipients [4–6] through a combination of damage to the vagus nerve causing dysfunction to the lower oesophageal sphincter and delayed gastric emptying [3].

Gastrointestinal physiology is also altered by immunosuppressive medications used following lung transplantation. Specifically, non-steroidal anti-inflammatory drugs, steroids, and cyclosporine A are all known to increase the rate of gastrointestinal ulceration [7], whilst azathioprine and mycophenolate motefil slow down intestinal gastric cell regeneration [8]. Note has been made of a disproportionate association between lung transplant recipients with gastric ulcers reaching a diameter of larger than 3 cm in patients that received high-dose non-steroidal anti-inflammatory drugs, cyclosporine, and high-dose corticosteroids after lung transplantation [9]. Furthermore, the likelihood of opportunistic infectious oesophagitis through candida, herpes simplex virus, and cytomegalovirus is increased with immunosuppressive medication [10]. Symptoms of both peptic ulceration and oesophagitis frequently include dysphagia, nausea, vomiting, and abdominal pain, which in turn increase risk of reflux, aspiration, and BOS [7].

Whilst anti-secretary medications such as proton pump inhibitors have shown to confer benefit in lung transplant recipients by protecting against rejection [11], there is shown to be an ongoing marked inflammatory response of bronchial epithelium [12]. This is postulated to be due to failure to regulate pepsin and bile acid secretion and subsequent aspiration, leading to allograft dysfunction [13]. Conversely, ARS provides a physical barrier that can minimise gastric content aspiration.

Considering the threat that BOS poses to allograft longevity, the utilisation of anti-reflux surgery (ARS) has garnered attention as a potential therapeutic avenue to mitigate the adverse effects of GORD post-lung transplantation by creating a mechanical barrier to reflux. To date, there have been many studies demonstrating improvements or at least stabilisation of lung function [13–26], in lung transplant recipients following ARS. Additionally, surgical complication rates in this demographic have been shown to be less than 5% [27].

Notwithstanding, the level of evidence supporting ARS in lung transplant patients is low with no published long-term prospective randomised controlled trials of ARS in lung transplant recipients. The dearth of high-quality evidence is quoted in a statement released by the ISHLT, in which emphasis is placed on consideration of ARS because of the importance of preservation of lung function, rather than high-quality evidence [27].

This paper therefore aims to provide a comprehensive review of the current literature surrounding the use of ARS and minimally invasive techniques following lung transplantation. By synthesising the available evidence, we aim to establish the survival benefit and allograft function in lung transplant patient associated with ARS. Moreover, we aim to identify gaps in knowledge and areas requiring further investigation that would ultimately help with the advancement of clinical practice and decision-making in the realm of ARS following lung transplantation.

## Methods

This systematic review was conducted according to a registered protocol and is reported in accordance with the Preferred Reporting Items for Systematic Reviews and Meta-Analyses (PRISMA) statement [28]. The review was registered on PROSPERO Centre for Reviews and Dissemination (registration number CRD42022379748).

### Search strategy

An electronic search was conducted out of MEDLINE, Embase, and the Cochrane library databases from inception until 13/01/2024 in addition to manual searches of selected articles. Two authors independently screened and reviewed relevant studies, with discrepancies resolved by a third author. The exact search strategy used is provided in Table 3.

### Inclusion and exclusion criteria

The inclusion and exclusion criteria were defined before starting the literature search. The following criteria were used for inclusion in the current review:

1. Population: patients all ages who were recipients of unilateral or bilateral lung transplant.
2. Intervention: patients who had undergone any anti-reflux procedure (fundoplication, magnetic sphincter augmentation, radiofrequency ablation) after lung transplant for all indications.
3. Comparison: no surgical or endoscopic procedure, or medical therapy only.
4. Outcome: reported outcomes of interest including lung function (FEV1, FVC, rate of change of FEV1), survival, and data pertaining to altered inflammatory mediators or microbiota.
5. Study Types: Randomised controlled trials (RCT), prospective or retrospective cohort studies, case (control) studies, and cross-sectional studies.

All non-human and non-English studies, reviews, published abstracts, conference presentations, single-case reports, editorials, and unpublished studies were excluded.

### Data extraction and quality assessment

A standardised data extraction form was developed on COVIDENCE, and two authors independently extracted all relevant data: study design, patient demographics, pre-treatment pH manometry testing, indication for ARS, pre-op work-up of gastro-oesophageal reflux disease (GORD), procedure, treatment of control groups, duration of treatment, and study outcomes. Any disagreements in data extraction between these two reviewers was resolved by a third reviewer. All the included studies were non-randomised, thus the Newcastle–Ottawa Scale (NOS) was used to evaluate the quality and Egger’s test was used to assess for potential publication bias of all included studies.

### Outcomes

The primary outcome of this review was measures of lung function, measured by forced expiratory volume in one second (FEV1), percentage of predicted FEV1 (%FEV1), and FEV1 rate of change. Secondary outcomes included measures of survival. For lung function outcomes, the effect measure calculated was mean difference. For survival, outcome measures calculated were hazard ratio and survival rates at defined time points. All results that were compatible in these outcome domains were included in the review.

Baseline characteristics including number of patients, age, study type, indication for lung transplant, pre-operative GORD work-up, and the time interval between lung transplant and ARS were also collected.

### Statistical analysis

Statistical analysis was performed using Stata Software, version 15.1 (StataCorp). Random effect analysis was used to calculated weighted mean differences and mass effect with associated 95% confidence intervals. This data was pooled using a random effects model and analysed using a random effects model (DerSimonian and Laird).

Data did not require preparation for synthesis; thus, no conversions were conducted. Data syntheses included in quantitative analysis is displayed in forest plots, and data used in qualitative analysis was displayed in data tables when appropriate.

Statistical heterogeneity was calculated using I^2^, where I^2^ < 30 was considered low, 30–60 moderate, and > 60 high heterogeneity. The effect size was illustrated in the form of forest plots accompanied with the calculated heterogeneity statistic (I^2^).

## Results

### Study selection

**Fig. 1:**
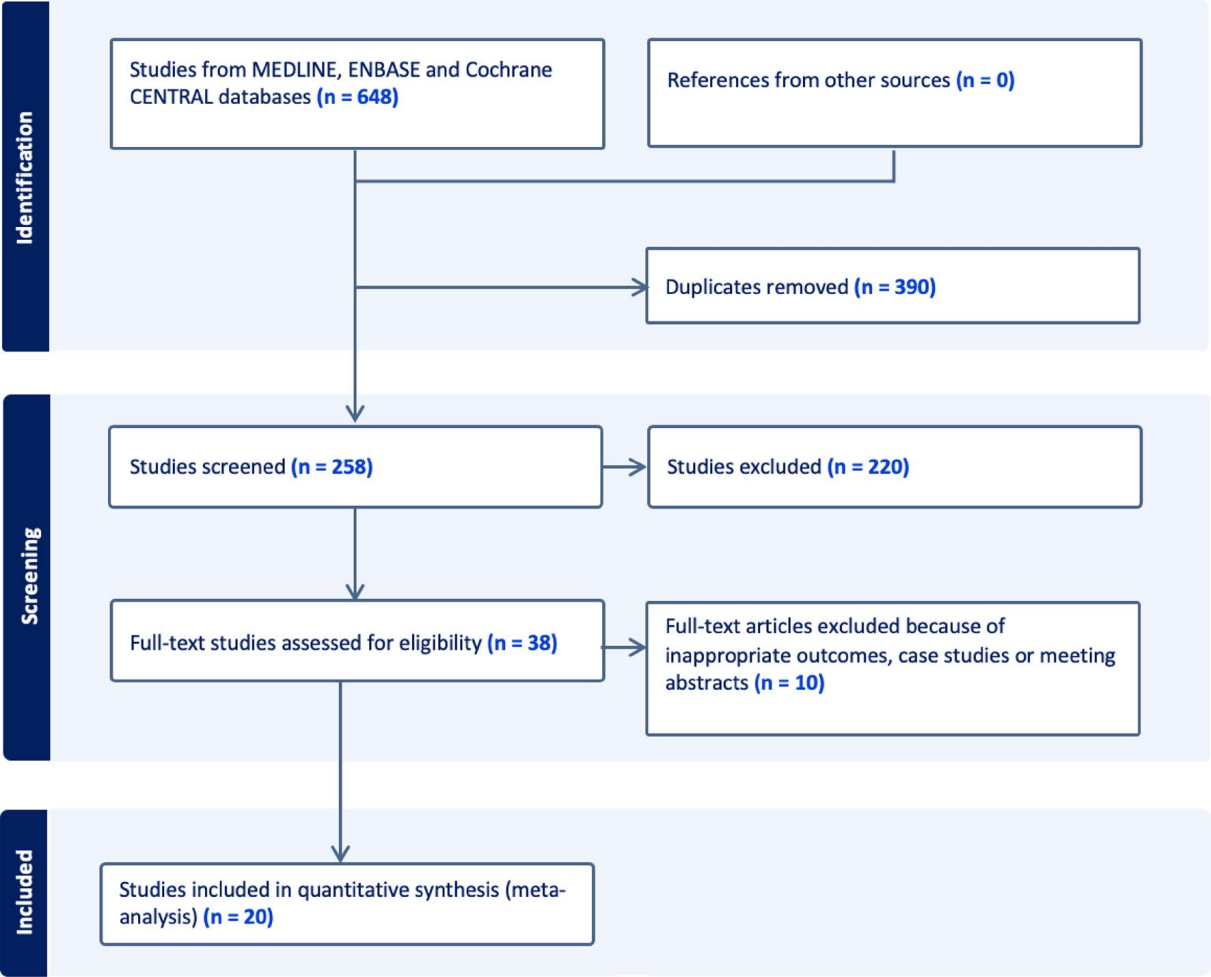
Flowchart of article screening and inclusion in accordance with Meta-analysis Of Observational Studies in Epidemiology guidelines. Six hundred and forty-eight studies were identified in the initial search, after exclusions, 38 were assessed for eligibility. 20 studies provided appropriate data to be analysed in the quantitative synthesis

The Meta-analysis of Observational Studies in Epidemiology guidelines for meta-analysis and observational studies were followed. The results of these are shown in Fig. 1. Six hundred and forty-eight studies were identified in the initial search, after exclusions, 38 were assessed for eligibility. 20 of these studies met the criteria to be included in the quantitative synthesis. Of the 38, 10 reported outcomes that were not appropriate for the current meta-analysis and a further 8 were excluded as they reported outcomes that could not be pooled with any other results.

### Study characteristics

A total of 1011 patients were included in the quantitative synthesis. Average age of patients ranged from 28 to 62, except for two studies that investigated paediatric populations and had an average age of nine and 14 [15, 29]. The vast majority of studies were retrospective cohort studies, besides three retrospective case series [25, 29, 30] and two prospective cohort studies [14, 31]. The average quality of studies according to the Newcastle–Ottawa Scale was 8.15.

The most common indications for lung transplantation were cystic fibrosis and obstructive lung diseases with at least 241 and 232 lung transplantations, respectively. Other indications included sarcoidosis, pulmonary hypertension, Alpha-1 antitrypsin deficiency, and chronic lung allograft dysfunction.

Indications for anti-reflux procedures varied amongst studies. 605 (59.8%) of patients underwent ARS based on abnormal pH studies, whilst 11 (1.1%) patients required both abnormal pH studies and normal gastric-emptying studies and 152 (15.0%) underwent ARS based on either abnormal pH studies or OGD findings consistent with oesophagitis. 99 (9.8%) had ARS after both abnormal pH studies and histological evidence of reflux following bronchoscopy and 16 (1.6%) after either abnormal pH studies or histological evidence after bronchoscopy. 21 (2.1%) patients were deemed to be candidates for ARS following abnormal results in 24-h pH studies, OGD, or bronchoscopy and 46 (4.5%) patients underwent ARS following a surgeon’s decision based on surgical history, symptoms, and oesophageal function (Table 1).

**Table 1:** Summary table of all studies included in quantitative analysis

	Number of patients	Average Age (range)	Type of study	Indication for lung transplant	Indication of ARS	Surgical Technique	Pre-op work-up for GORD	Average timing of ARS relative to Lung Transplant (with SD)
Abbassi-Ghadi et al. [13]	38	46.6 (20–67)	Retrospective Cohort	COPD (*n* = 14), CF (*n* = 9),PH (*n* = 1), Sarcoidosis (*n* = 3), Other (*n* = 8)	Abnormal pH studies with histological evidence of gastroesophageal reflux aspiration on bronchoscopy	Nissen (all)	pH Impedance; Bronchoscopy	25.2 ± 11.3 months
Biswas Roy et al. [21]	86	62.5 (54–66)	Retrospective Cohort	Obstructive Lung Disease (*n* = 53), Restrictive lung disease (*n* = 23), CF (*n* = 7), Re-transplantation (*n* = 1), Other (*n* = 2)	Abnormal pH studies	37 Toupet, 47 Nissen, 1 Dor. Wrap type depended on oesophageal motility studies	pH Impedance Manometry; DeMeester	8.7 ± 7.5 months
Burton et al. [32]	21	43 (20–68)	Retrospective Cohort	CF (*n* = 15); PH (*n* = 1); COPD (*n* = 1); PF (*n* = 2); A1AT (*n* = 1)​	Symptomatic GORD in combination with abnormal results in one of OGD, 24-h pH studies or positive aspiration on bronchoscopy	16 Toupet; 5 Nissen. Fundoplication protocol changed from Nissen to Toupet halfway through study	pH Impedance; bronchoscopy; DeMeester	22.5 ± 20.9 months
Cantu III et al. [23]	76	43 (no range)	Retrospective Cohort	COPD/A1AT (*n* = 26); CF/Bronchiectasis (*n* = 29); IPF (*n* = 15); BO/Retransplant (*n* = 2); PPH (*n* = 2); Other (*n* = 2)​	Abnormal pH studies	77 Nissen; 5 Toupet; 3 Belsey Mark IV​. Borderline oesophageal clearance underwent Toupet	pH Impedance, Manometry	18.6 ± 20.6 months
Davis Jr et al. [19]	43	43.7 (16–66)	Retrospective Cohort	CF/bronchiectasis (*n* = 41), COPD/ A1A def (*n* = 51), BO/Rtx (*n* = 2), IPF (*n* = 9), PH (*n* = 2), other (*n* = 7)​	Abnormal pH studies in patients with symptomatic GORD or unexplained decrease in FEV1 (*n* = 39), barium swallow with normal manometry (*n* = 2), repetitive aspiration (*n* = 2)	36 Nissen, 4 Toupet	pH Impedance; barium swallow; manometry	–
Jamie Dy et al. [15]	11	14.3 (1–21)	Retrospective Cohort	CF (*n* = 15); BOS (*n* = 4); PH (*n* = 7); Pulmonary vein stenosis (*n* = 1); PF (*n* = 2); Surfactant deficiency (*n* = 1)	Abnormal pH studies with symptomatic reflux and normal GES	Nissen (all)	pH impedance; Gastric-Emptying Studies	9.7 ± 5.7 months
Frankel et al. [35]	143	56.2 (IQR 44.1–65.7)	Retrospective Cohort	CF (*n* = 53); COPD (*n* = 50); IPF (*n* = 25); Other (*n* = 21)	Abnormal pH studies in those with symptomatic GORD and/or CLAD with no other cause identified	Toupet (all)	pH impedance	17 ± 7.3 months
Green et al. [18]	136		Retrospective Cohort​	COPD (*n* = 36), IPF (*n* = 55), CF (*n* = 33), PH (*n* = 1), Sarcoidosis (*n* = 3), Other (*n* = 8)​	Abnormal pH studies	120 Nissen; 15 Toupet; 1 Dor​	pH impedance	2.8 ± 1.1 months
Halpern et al. [22]	17	61 (IQR 44–71)	Retrospective Cohort	–	Abnormal pH studies; barium swallow; bronchoscopic evidence of aspiration in symptomatic individuals	LINX (*n* = 17); Fundoplication (Not specified) (*n* = 17)	pH impedance, manometry, barium swallow; bronchoscopy	16.8 ± 19.6 months
Hoppo et al. [33]	22	53 (21–70)	Retrospective Cohort	COPD (*n* = 11), IPF (*n* = 14), CF (*n* = 6), scleroderma (*n* = 7), others (*n* = 5)​	Abnormal pH studies in those with symptomatic GORD or declining lung function with no other cause identified	24 Nissen; 17 Dor​. Dor fundoplication for those with severe oesophageal dysmotility	pH impedance; OGD; barium swallow; manometry	31 ± 24 months
Kolbeinsson et al. [25]	11	61 (28–70)	Retrospective Case-Series	COPD (*n* = 5), Bronchiectasis (*n* = 2), IPF (*n* = 2), CF (*n* = 1), A1AT (*n* = 1)	Abnormal pH studies in those with symptomatic GORD and failed medical management	Stretta (all)	pH impedance	11 ± 5.3 months
Kowalski et al. [39]	53	–	Retrospective Cohort	COPD/Emphysema (*n* = 19), IPF (*n* = 18), CF (*n* = 8), PF (*n* = 4), A1AT deficiency (*n* = 1), Bronchiectasis (*n* = 1), BO (*n* = 1), PH (*n* = 1)​	Abnormal pH studies—moderate to severe reflex (defined at DeMeester score > 28)​	48 Toupet; 5 Dor. Toupet and Dor reserved for poor motility	pH impedance; OGD; manometry; DeMeester	13.2 ± 17.5 months
Lau et al. [26]	18	39 (19–66)	Retrospective Cohort	CF (*n* = 7), COPD (*n* = 6), PH (*n* = 1), IPF (*n* = 1), Other (*n* = 3)​	Abnormal pH studies	14 Nissen; 4 Toupet	pH impedance; manometry; Gastric-Emptying Studies	–
Leiva-Juarez et al. [16]	10	52 (50–62)	Retrospective Cohort	Systemic Sclerosis (*n* = 10)	Abnormal pH studies	5 Dor; 4 Nissen; 1 Toupet​. Based on manometry/GES	pH impedance; Manometry; Gastric-Emptying Studies	16.4 ± 11.5 months
Leiva-Juarez et al. [17]	152	–	Retrospective Cohort	ILD, COPD, AAD, CF, other	Abnormal pH studies with DeMeester score > 14.72 or OGD findings consistent with reflux oesophagitis)​	117 Nissen; 8 Dor; 2 Toupet; 1 Belsey Mark IV. Surgeon’s discretion. Nissen unless contraindicated​	pH impedance; OGD	–
Pegna et al. [24]	57	41 (16–61)	Retrospective Cohort	–	Symptomatic reflux or abnormal pH studies	Nissen (all)	pH impedance	–
Razia et al. [20]	46	61.9 (55.6–68.1)	Retrospective Cohort	Obstructive Lung Disease (*n* = 19), Pulmonary vascular disease (*n* = 1), CF (*n* = 3), restrictive lung disease (*n* = 2), PF (*n* = 2)	Abnormal pH studies and/or an unexplained decline in pulmonary function	Toupet (all)	pH impedance; manometry; Gastric-Emptying Studies; barium swallow	5.9 ± 1.1 months
Robertson et al. [14]	16	38.2 ± 11.9	Prospective Cohort	CF (*n* = 10), COPD/Asthma (*n* = 1, COPD (*n* = 1), PF (*n* = 3), PF/asthma (*n* = 1)​	Abnormal pH studies in symptomatic individuals, those with deteriorating lung function or suspected microaspiration	Nissen (all)	pH impedance; manometry; OGD; bronchoscopy	34.6 ± 28.9 months​
Yergin et al. [31]	44	56.5 (48/65)	Prospective Cohort	COPD (*n* = 18); ILD (*n* = 18), CLAD/BOS (*n* = 5); CF (*n* = 4); A1AT (*n* = 3)	Acute cellular rejection or documented amylase or pepsin on bronchoscopy in those with abnormal pH studies and symptomatic individuals	Toupet (all)	pH impedance; Barium swallow; Gastric-emptying studies; Manometry	12.9 ± 3.6 months
Zheng et al. [29]	11	9.6 (5.6–15.9)	Retrospective Case-Series	Radiation-induced pulmonary fibrosis (*n* = 2), emphysema (*n* = 1), pulmonary hypertension (*n* = 6), cystic fibrosis (*n* = 1)	Abnormal pH studies	Nissen (all)	pH impedance; gastric-emptying; OGD	14.0 ± 18.6 months

*CF* cystic fibrosis, *COPD* chronic obstructive pulmonary disease, *PH* pulmonary hypertension, *IPF* idiopathic pulmonary fibrosis, *PF* pulmonary fibrosis, *A1AT* alpha-1 antitrypsin deficiency, *ILD* interstitial lung disease, *ALD* alveolar lung diseases, *BO* bronchiolitis obliterans, *OGD* Oesophagogastroduodenoscopy

The most common anti-reflux performed was Nissen’s fundoplication followed by Toupet’s fundoplication with 578 and 365 procedures documented, respectively. One study investigated the use of the LINX device [22] and one study investigated the Stretta procedure [25]. Average time from lung transplant to ARS varied in studies, with an average of 2.8 to 34.6 months (Table 1).

Post-operative rates of infection were reported in 4 studies and varied between 0 and 20% [16, 22, 32, 33]. Prophylactic antibiotic regimens were described in two studies [19, 29]. 3 studies involved routine monitoring of patients in a high dependency unit or intensive care unit [13, 24, 33], whilst 3 studies admitted planned to admit patients to higher level care if they were deemed to be medically complex preoperatively [14, 32, 33].

### Changes in FEV1, % FEV1, and rate of change of FEV1 following ARS

Thirteen studies published results of lung function tests on patients’ post-lung transplant that had anti-reflux surgery. Eight studies reported FEV1 (L/s) pre- and post-ARS, eight reported on %FEV1, and six studies reported on the rate of change (RoC) of FEV1 (mL/d). Overall, lung function data were extracted from 504 patients. Eleven of these patients underwent a STRETTA procedure and the remainder underwent fundoplication (Table 1).

**Fig. 2:**
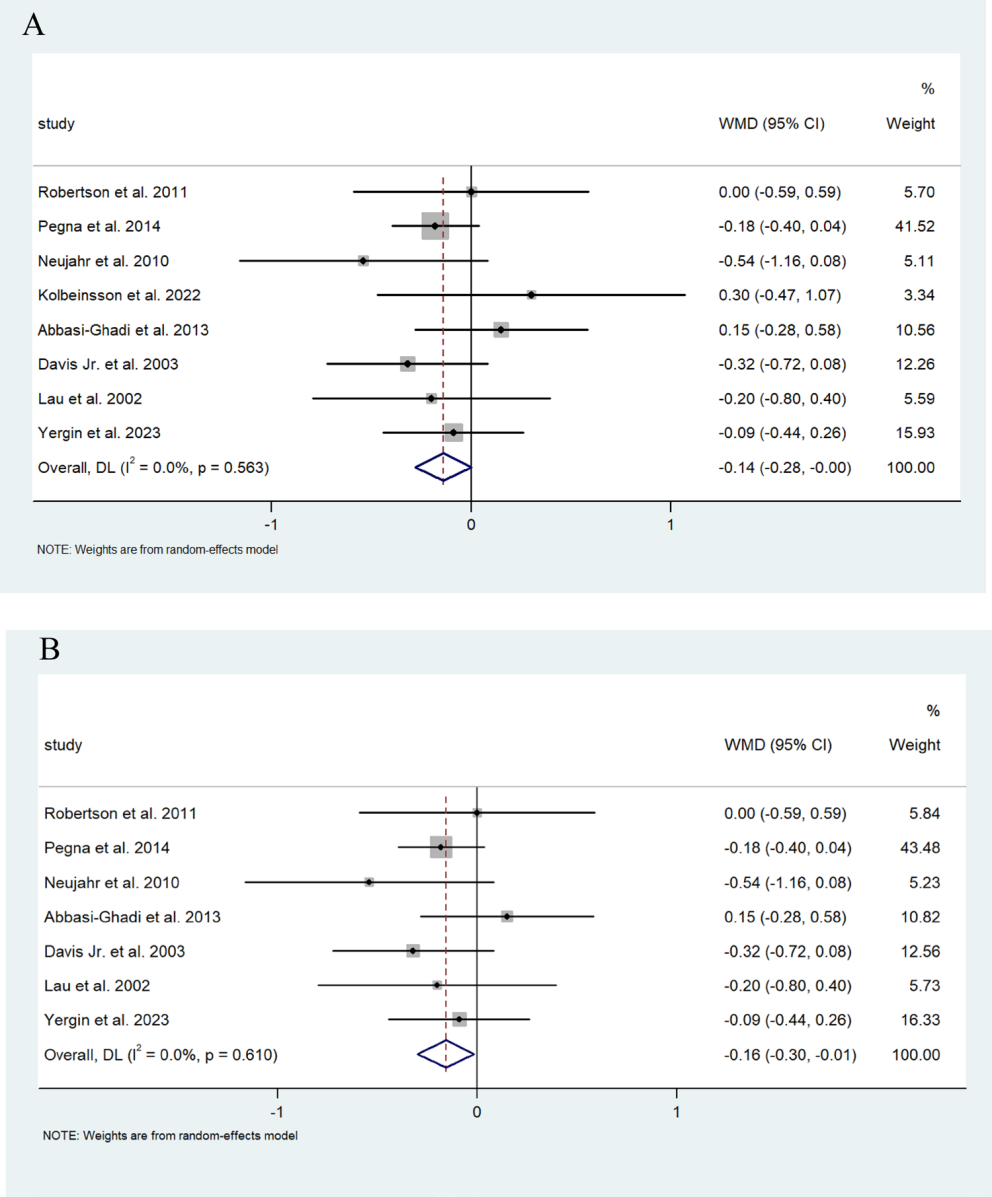
**A** Forest plot demonstrating the mean weighted difference (MWD) in FEV1 (L/s) in patients after lung transplant undergoing ARS. Effect size is plotted to permit comparison of studies reporting FEV1 values before and after ARS. **B** Forest plot demonstrating the MWD in FEV1 (L/s) in patients after lung transplant undergoing fundoplication. Effect size is plotted to permit comparison of studies reporting FEV1 values before and after fundoplication

Random effect analysis for FEV1 (L/s) from 477 patients demonstrated a 0.141 L/s (95% CI 0.001, 0.282) improvement following ARS (I^2^ = 0.0%) and 462 of these patients underwent fundoplication and showed a 0.157 L/s improvement (95% CI −0.014, −0.157) ARS (I^2^ = 0.0%) (Fig. 2A, B).

**Fig. 3:**
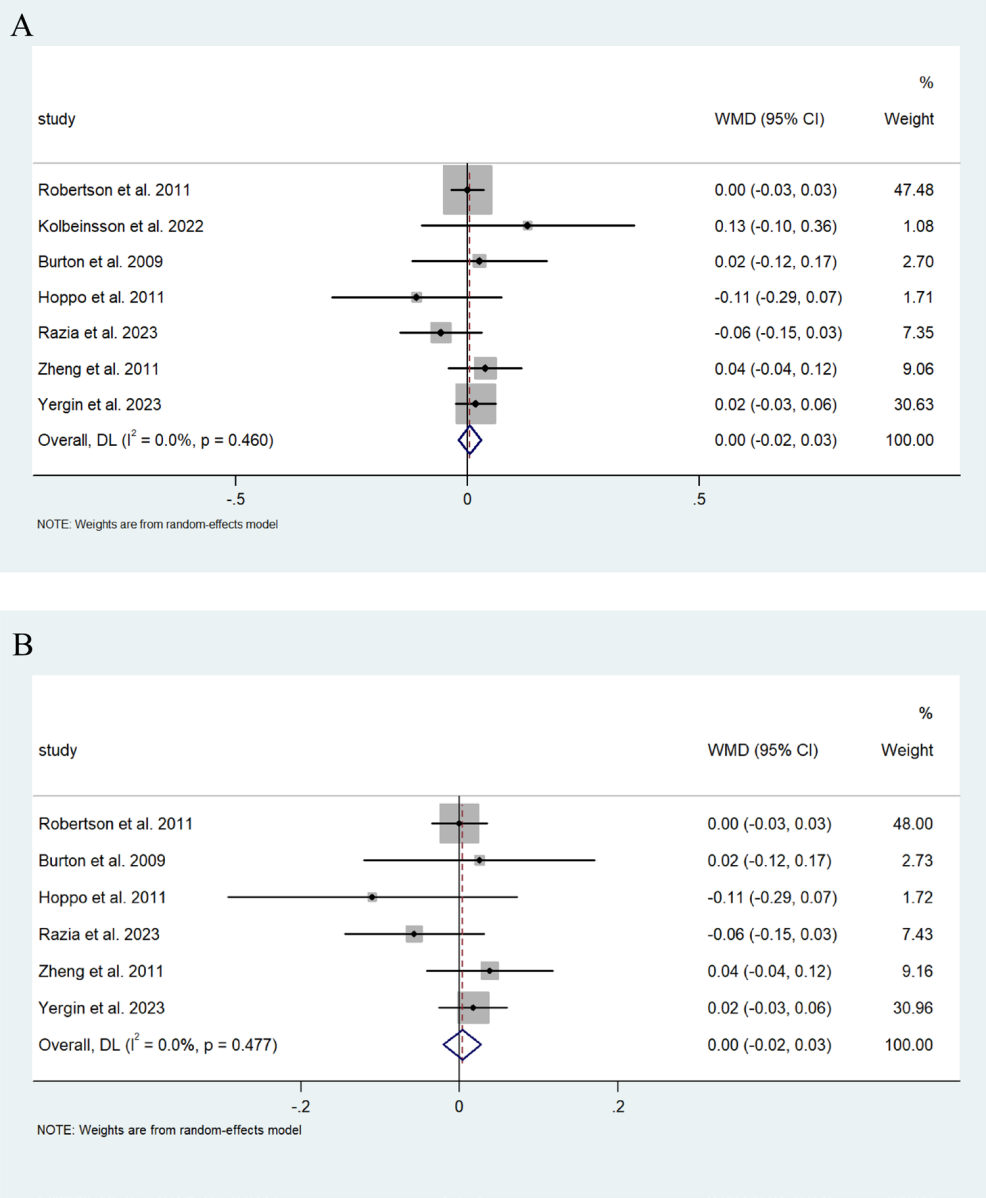
**A** Forest plot demonstrating the MWD in %FEV1 of predicted FEV1 in patients after lung transplantation undergoing ARS. Effect size is plotted to permit comparison of studies reporting FEV1 values before and after ARS. **B** Forest plot demonstrating the MWD in FEV1% of predicted FEV1 in patients after lung transplantation undergoing fundoplication. Effect size is plotted to permit comparison of studies reporting FEV1 values before and after fundoplication

Random pooled effect analysis for FEV1% data from 364 patients that underwent ARS demonstrated a very modest decrease of 0.005% (95% CI; −0.019, 0.029) (I^2^ = 0.0%) of FEV1%. Similarly, the 320 patients of this group who had undergone fundoplication had a decrease of 0.003% of FEV1% (95%CI −0027, 0.021) (I^2^ = 0.0%) (Fig. 3A, B).

**Fig. 4:**
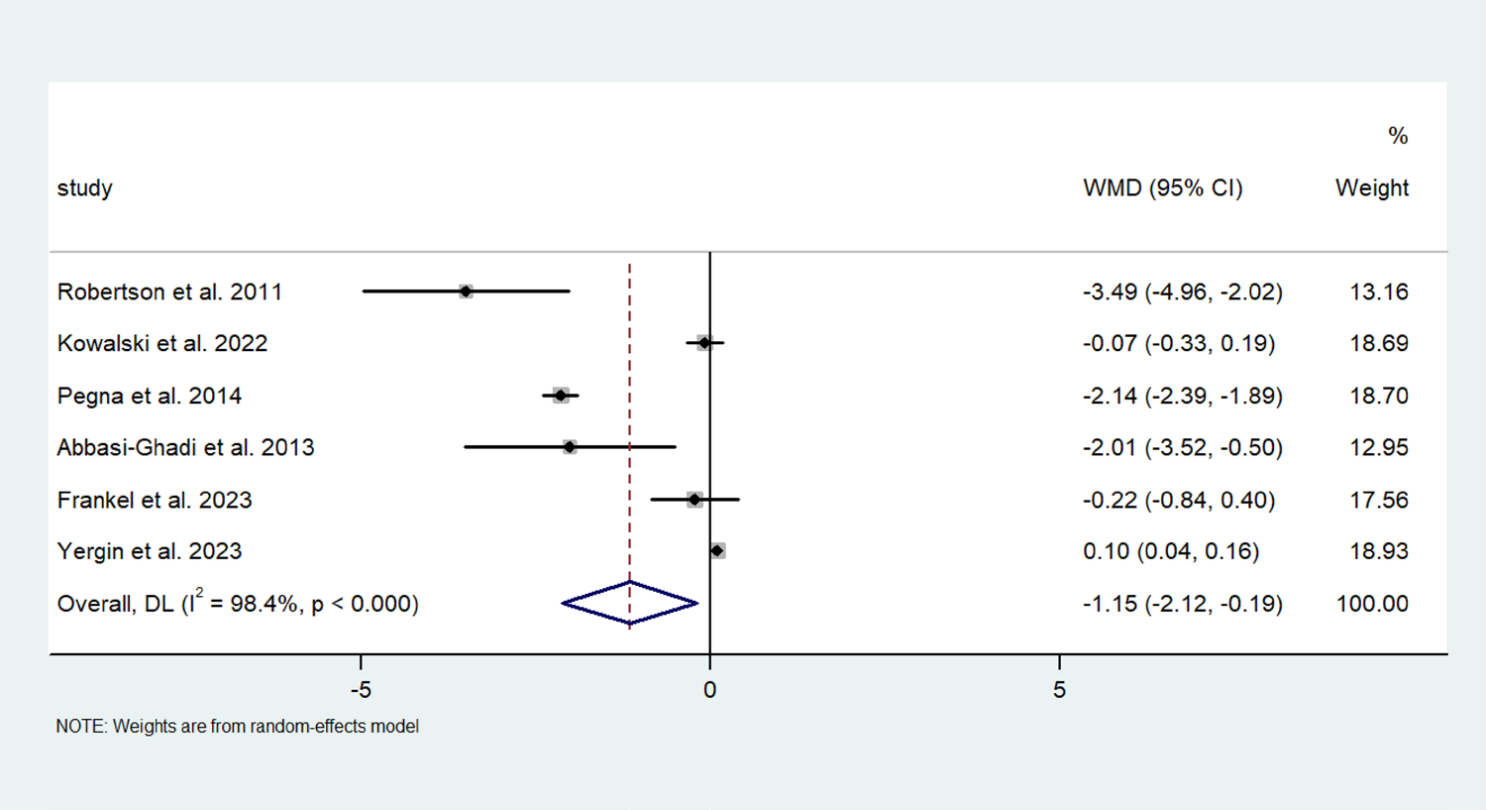
Forest plot demonstrating the MWD of the rate of change of FEV1 (mL/d) in patients after lung transplantation undergoing ARS. Effect size is plotted to permit comparison of studies reporting FEV1 values before and after ARS

Random pooled effect analysis on the rate of change of FEV1 from 351 patients who all underwent fundoplication showed a decrease of −1.153 mL/d (95% CI; −12.117, −0.188) in the rate of change of FEV1. Although individually strong effect sizes were proven in three of the six studies, there was significant heterogeneity noted (I^2^ = 98.4%) (Fig. 4).

### Survival following ARS

There were nine studies reporting on survival following ARS and lung transplant surgery, in total this included data from 577 patients. Five studies which included 309 patients published data on survival hazard ratios, four studies on 5-year survival, and 6 more generally on medium-term survival, which was established to be between one and five years for the purposes of this study. The number of patients included for the meta-analysis for 5-year survival and medium-term survival were 185 and 295, respectively. Seventeen patients included in the medium-term survival analysis underwent magnetic sphincter augmentation with the LINX device, the remainder of the patients included in the survival data underwent a fundoplication.

**Fig. 5:**
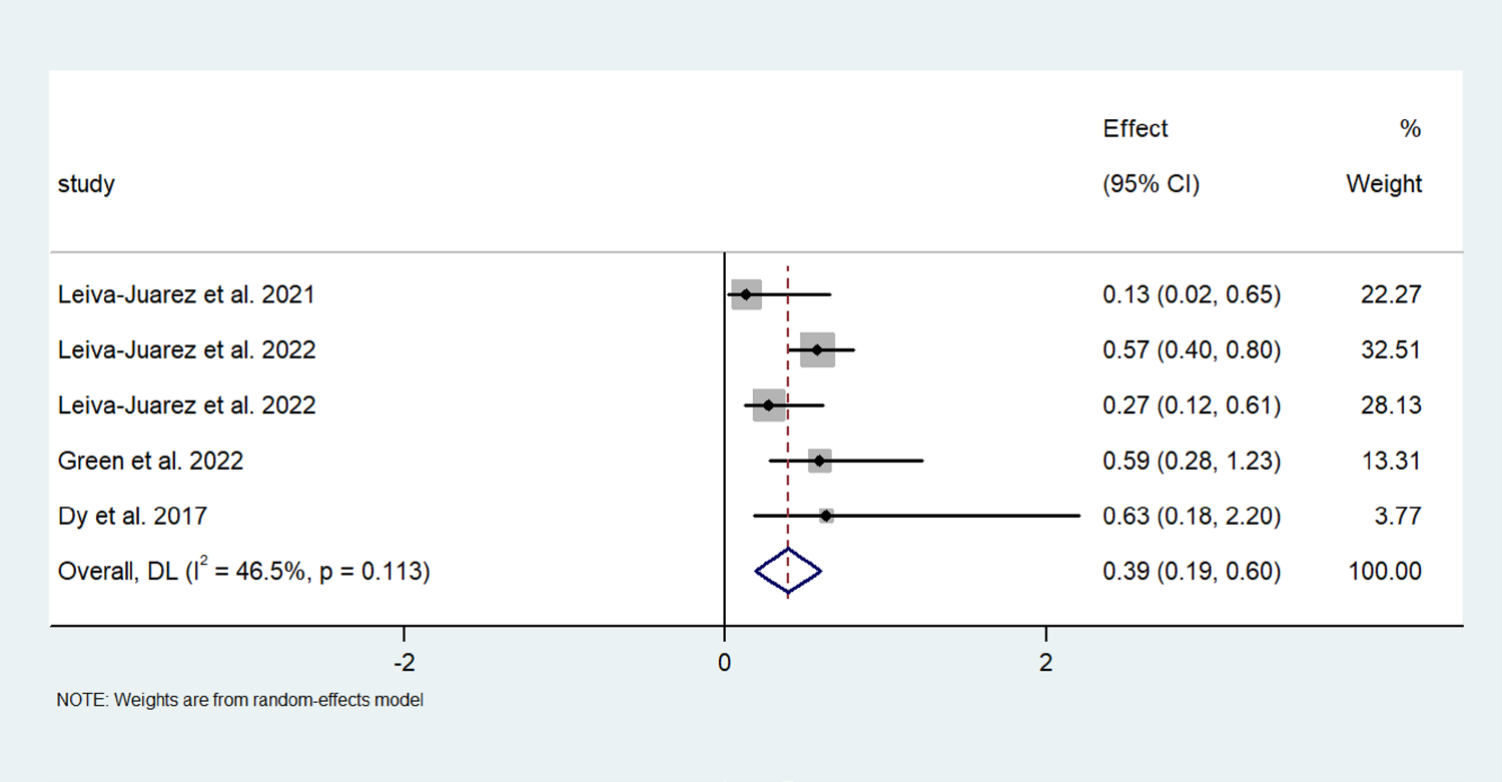
Forest plot demonstrating the effect size of ARS on the survival hazard ratio in lung transplant patients

**Fig. 6:**
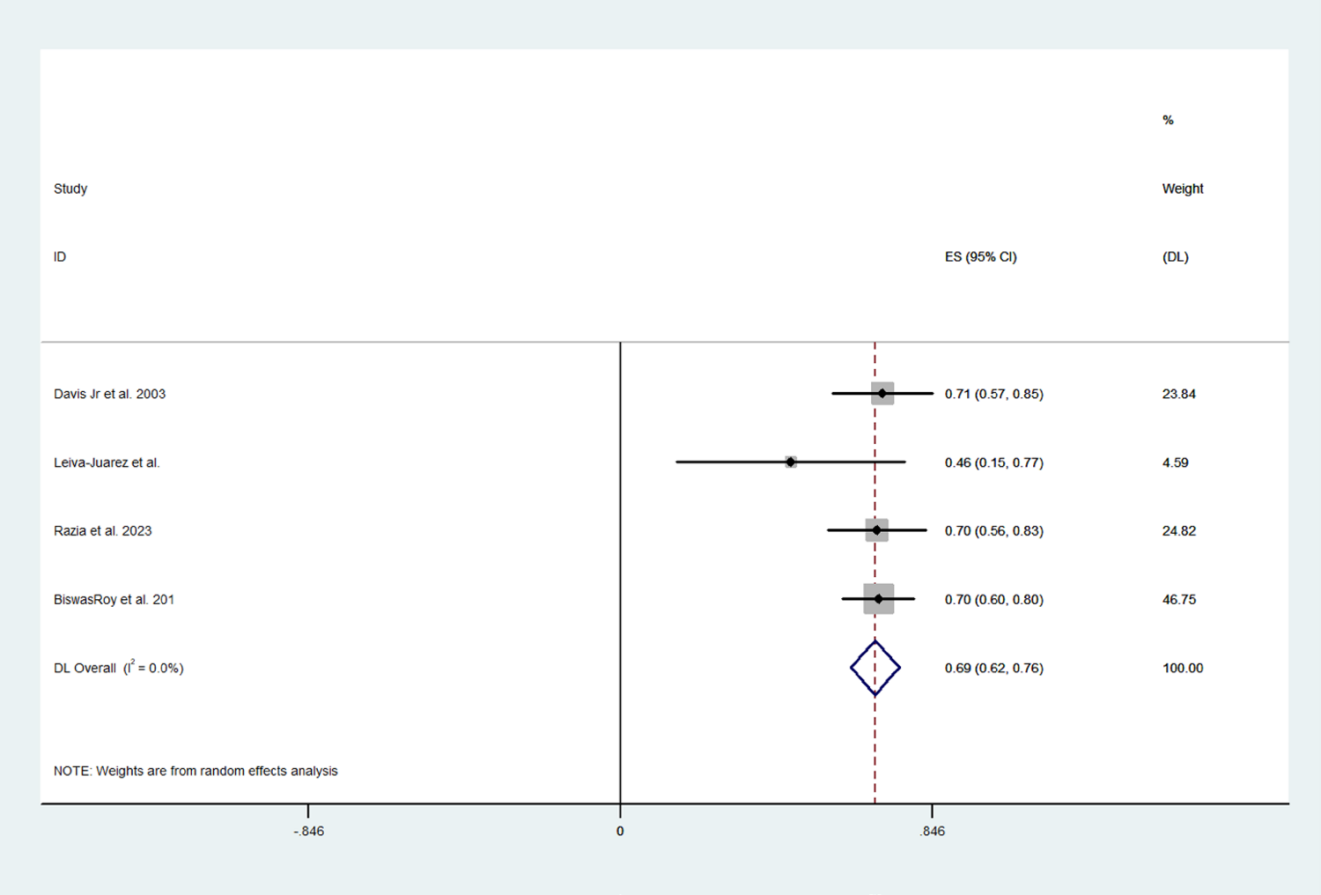
Forest plot demonstrating the effect size of ARS on 5-year survival rates following lung transplantation

**Fig. 7:**
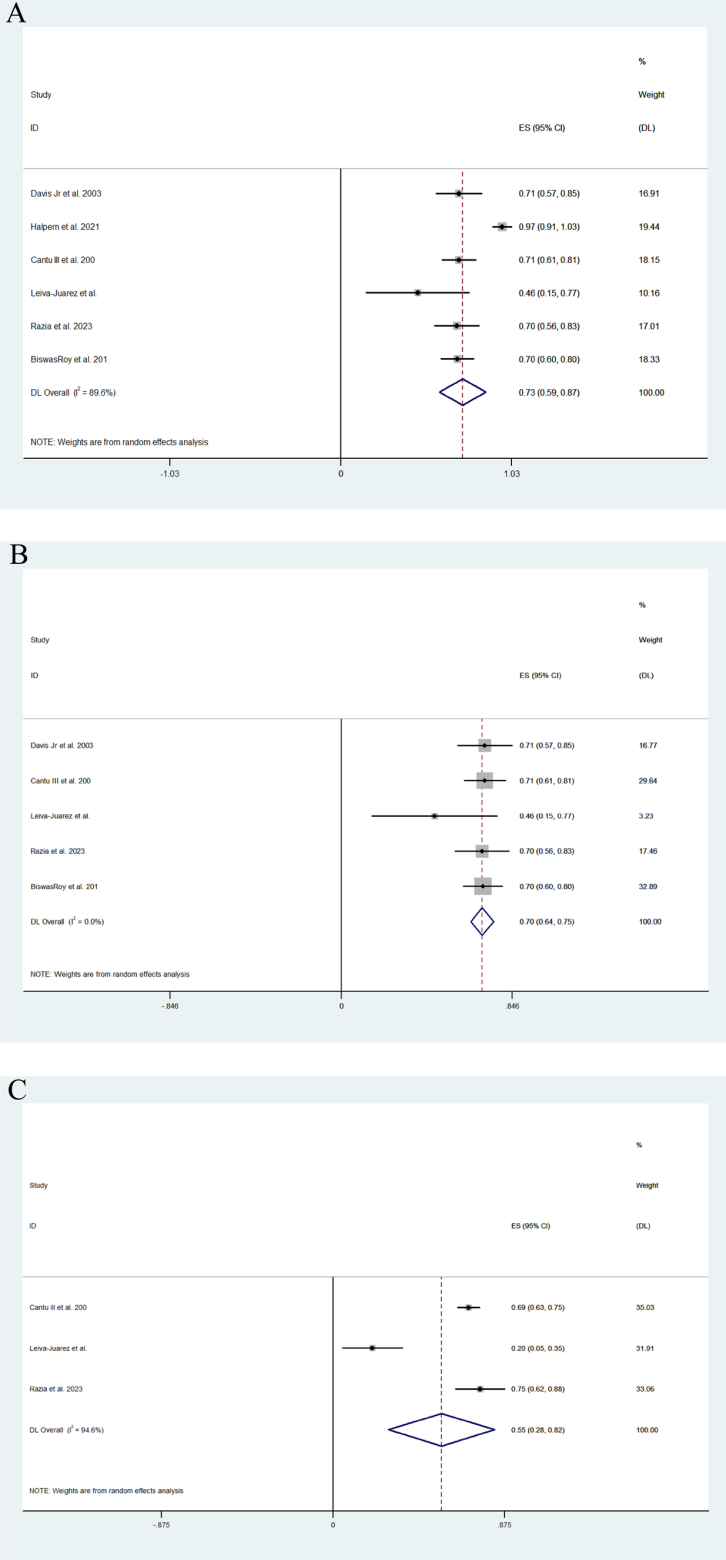
**A** Forest plot illustrating medium-term survival in patients with ARS post-lung transplantation. **B** Forest plot illustrating medium-term survival in patients after fundoplication post-lung transplantation. **C** Forest plot illustrating medium-term survival in patients following lung transplantation with no ARS

Pooled random effect analysis investigation multivariate survival hazard ratios revealed a hazard ratio of 0.39 (95% CI; 0.19, 0.60) in patients following lung transplantation (I^2^ = 46.5%) (Fig. 5). Moreover, 5-year survival was greater following ARS, and the effect size was 0.69 (95% CI; 062, 0.76) (I^2^ = 0.0%) (Fig. 6). Medium-term survival was investigating by analysing ARS, which included the use of the LINX device and fundoplication and control studies independently to achieve greater power. Effect size was larger for the ARS group than in the control group (0.731 (95% CI; 0.591, 0.871) vs 0.553 (95% CI; 0.283, 0.824)) (I^2^ = 89.59%). Medium-term survival following fundoplication was similar to that of the fundoplication and LINX group, with the effect size of the fundoplication group being 0.696 (95% CI 0.641–0.752). Large heterogeneity was observed between the included studies investigating multivariate survival hazard ratios (I^2^ = 94.64%) (Fig. 7A–C).

### Comparison of fundoplication techniques

There were 7 studies in which the Nissen fundoplication was the preferred surgical method which included 205 patients, of which 197 underwent a Nissen fundoplication. In comparison, there were 5 studies which included 307 patients, of which 297 underwent a Toupet fundoplication.

**Fig. 8:**
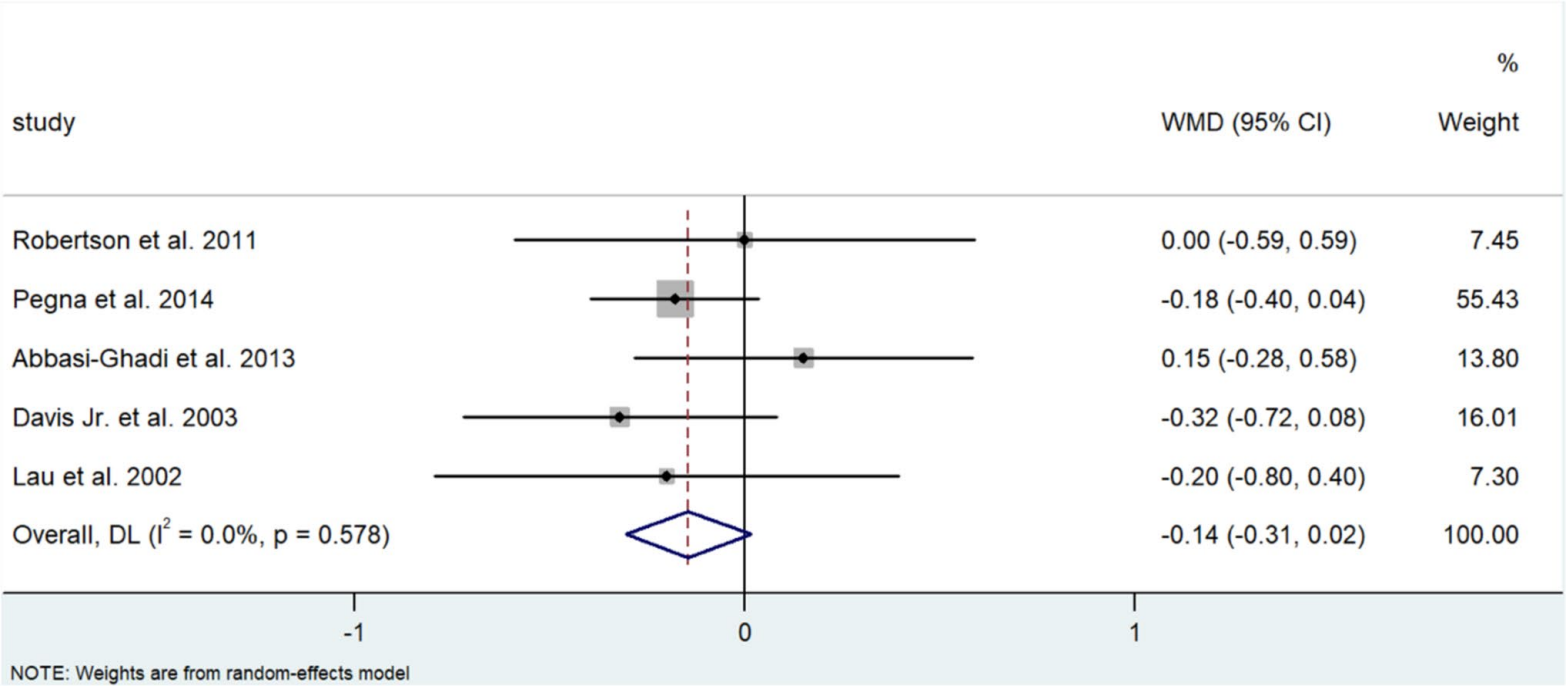
Forest plot illustrating the changes in the WMD of FEV1 (L/s) in patients after lung transplantation undergoing Nissen fundoplication. Effect size is plotted to permit comparison of studies reporting FEV1 values before and after fundoplication

**Fig. 9:**
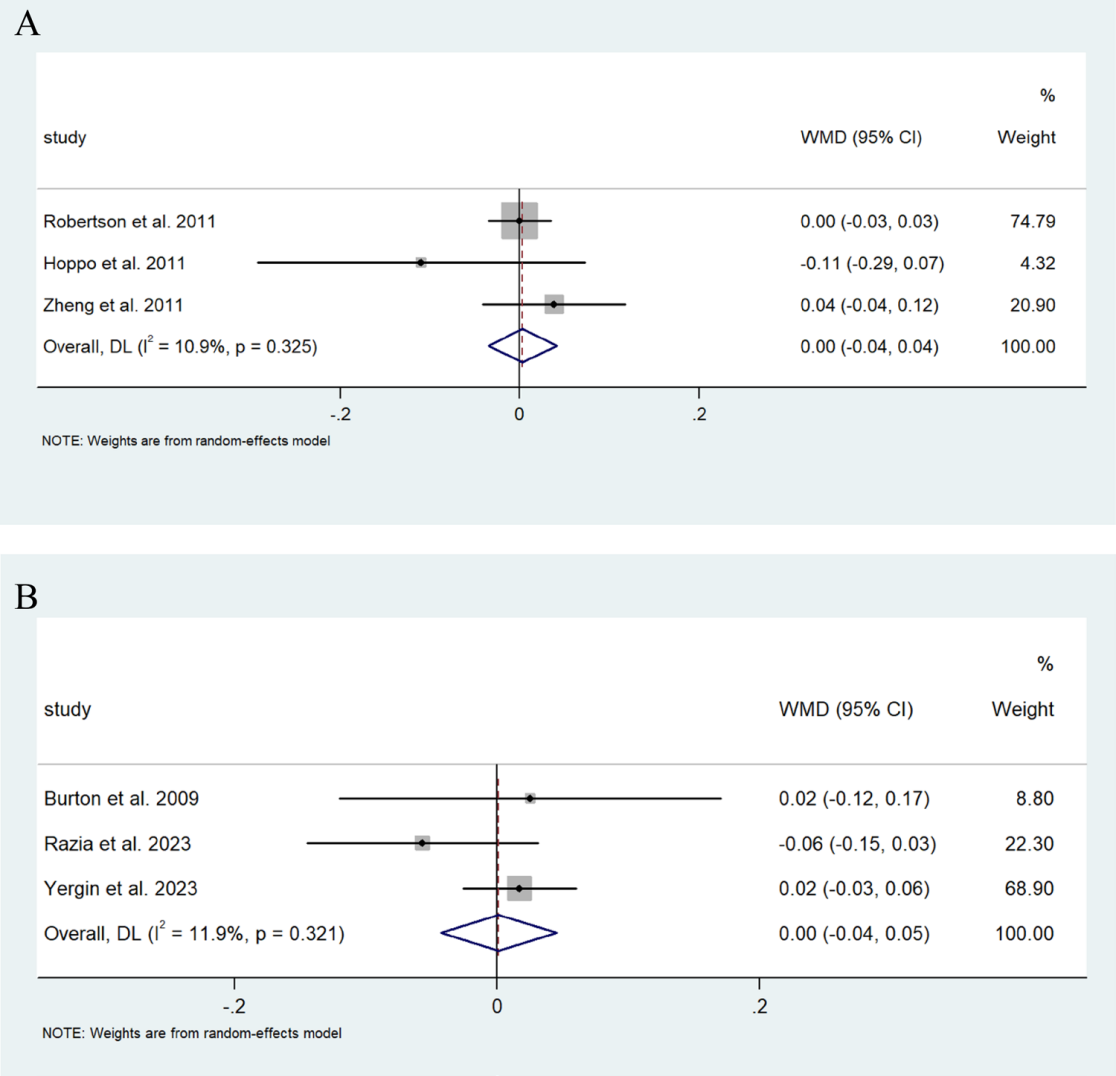
**A** Forest plot illustrating changes in the WMD of %FEV1 of predicted FEV1 in patients after lung transplantation undergoing Nissen fundoplication. Effect size is plotted to permit comparison of studies reporting %FEV1 values before and after fundoplication. **B** Forest plot illustrating changes in the WMD of %FEV1 in patients after lung transplantation before and after Toupet fundoplication. Effect size is plotted to permit comparison of studies reporting %FEV1 values before and after fundoplication

**Fig. 10:**
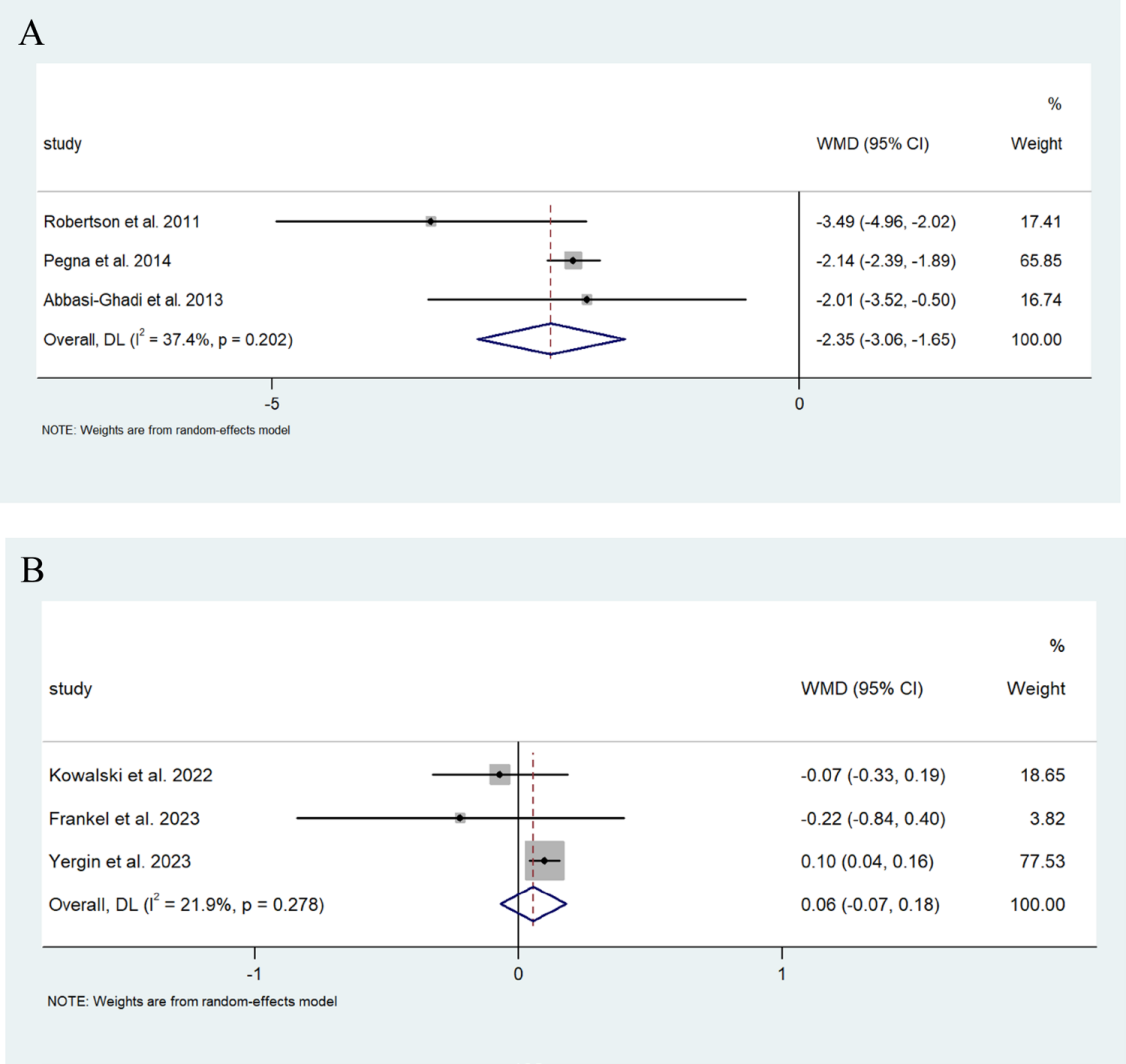
**A** Forest plot demonstrating the WMD in the ROC of FEV1 (mL/d) in patients after lung transplantation before and after Nissen fundoplication. Effect size is plotted to permit comparison of studies reporting rate of change in FEV1 values before and after fundoplication. **B** Forest plot demonstrating the WMD between the ROC of FEV1 (mL/d) in patients after lung transplantation before and after Toupet fundoplication. Effect size is plotted to permit comparison of studies reporting rate of change in FEV1 values before and after fundoplication

Pooled analysis revealed a similar increase in FEV1 in those that underwent a Nissen fundoplication in comparison to the single study that reported on FEV1 changes in which patients underwent a Toupet’s fundoplication [31] (0.125 L/s (95% CI; −0.016, 0.306) vs 0.09 L/s (95% CI; −0.262, 0.442)) (I^2^ = 0.0%) (Fig. 8). Changes in %FEV1 were also comparable in those that underwent a Nissen to those who underwent a Toupet fundoplication (−0.003% (95% CI −0.042, 0.035) vs −0.001% (95% CI −0.045, 0.043), respectively (I^2^ = 10.9%, 11.9%)) (Fig. 9A, B). Contrastingly, those who underwent a Nissen fundoplication had a greater decrease in the rate of change of FEV1 of −2.353 mL/d (95% CI; −3.058, −1.649), compared to 0.056 mL/d (95% CI; −0.068, 0.180) in those who underwent a Toupet fundoplication (I^2^ = 37.4%, 21.9%, respectively) (Fig. 10A, B).

## Discussion

This systematic review and meta-analysis demonstrates an improvement in FEV1 and the rate of change of FEV1 in lung transplant patients who have undergone ARS. Furthermore, survival hazard ratios, 5-year survival and medium-term survival had all improved in patients who had ARS in comparison to those who had not. Moreover, a comparison of fundoplication techniques demonstrated the greatest improvement in rate of decline of allograft function by measuring FEV1 per day in those undergoing a Nissen fundoplication.

To date, there has been one meta-analysis investigating lung function tests in lung transplant patients following ARS [34] which also demonstrates a statistically significant improvement in the rate of change of FEV1 (−2.12 mL/day pre-ARS vs 0.05 mL/day post-ARS, *p* < 0.0001) and a statistically non-significant change in FEV1 following ARS (2.02 vs 2.14, *p* > 0.05) [34]. In the present review, three of the six studies investigating the rate of change of FEV1 reported significant improvements following ARS, whilst the remaining studies did not demonstrate such improvements. Importantly, the studies that observed the most substantial benefits in FEV1 rate of change were those involving patients who exhibited the steepest declines in FEV1 prior to surgical intervention [13, 14, 24].

The absolute improvement of 0.141 L/s in FEV1 or modest decline of 0.005% in % FEV1 are unlikely to be of clinical significance. However, the slower rate of lung allograft decline as characterised by a 1.153 mL/d improvement in rate of change of FEV1 are an indication that chronic lung allograft disease (CLAD) progression can be stalled or decelerated following ARS. Given the immune and fibrotic nature of CLAD and it is association with GORD, it is plausible that ARS provides a physical barrier that may not be achieved by aggressive rehabilitation alone. It has been hypothesised that once chronic lung allograft is diagnosed that the decline in function cannot be reversed through ARS [35]. This notion is supported by various animal model studies, which have demonstrated that if an allogeneic organ has started to show signs of chronic rejection and is subsequently transplanted into a syngeneic host, injury will persist in the absence of ongoing alloimmune damage [36, 37].

It would therefore be advisable for ARS to be considered soon after lung transplant, rather than waiting for stagnation in FEV1 values. Published data indicate that early fundoplication is associated with slower long-term decline in lung function [21], greater freedom from BOS, as well as a lower risk of acute rejection compared to a late fundoplication [23, 38]. Previous data reinforces this finding, with the largest survival benefits of ARS in lung transplant patients observed in lung transplant patients who undergo ARS prior to CLAD diagnosis [17] and when done within 90 days of lung transplantation [23].

The indications for ARS varied across the studies reviewed. Some centres recommended ARS in lung transplant recipients based on declining pulmonary function tests and positive pH monitoring results, whereas others limited ARS to those with positive pH studies alone (Table 1). In one study there was a change in the selection of surgical candidates during the study. The authors observed a high prevalence of GORD and demonstrable improvements of FEV1 after fundoplication in lung transplant patients and subsequently introduced routine pH studies in all patients after lung transplant in the latter part of its study [19]. Similarly, Kowalski et al. [39] advocated for routine pH monitoring at 90-day intervals post-transplant, with symptomatic individuals referred for expedited investigations, including pH monitoring and manometry. The transition to routine early pH monitoring after lung transplantation suggests that both clinical and subclinical GORD can be identified and that timely ARS intervention for these patients may play an important role for allograft longevity. If widely adopted, this approach could potentially prevent CLAD and enhance long-term graft survival.

The studies reviewed in this meta-analysis also provide an insight into the use of fundoplication in the treatment and alleviation of GORD. Fundoplication consistently demonstrated high patient satisfaction and significant in GORD-related symptoms when assessed using validated questionnaires including the Carlsson reflux score, RSI (reflux symptom index), and GIQLI (gastrointestinal quality of life index) [14, 24, 29, 31, 32, 39]. Similarly, two studies observed a dramatic reduction in DeMeester score post fundoplication with the average score decreasing from 45.8 to 1.8 and 25.9 to 1.4 [31, 39]. A consistent decrease in the burden of GORD poses an additional benefit and consideration of ARS in lung transplant patients.

It has been theorised that patients with restrictive lung disorders have better survival outcomes than with obstructive lung diseases. It has been understood that this is due to differences in oesophageal motility profiles, in which restrictive disorders tend to have higher thoraco-abdominal pressure gradients and DeMeester score [40]. Following transplantation, a reduction in thoraco-abdominal pressure gradients and increased contractility result in more profound GORD [40, 41]. This is a finding that is reinforced by a study in this review which was associated with the lowest survival hazard ratio (0.13) and had solely investigated patients with systemic sclerosis-related lung disease [16].

Inflammatory markers have been analysed in bronchoalveolar lavage fluid (BALF) in 3 studies involving 34 patients undergoing fundoplication. After ARS, there was a demonstrated reduction in the frequency of CD8 lymphocytes, lymphocytes, neutrophils, and interleukins in BALF with a restoration of physiological levels of macrophages following ARS for lung transplant patients (Table 2) [30, 42, 43]. These inflammatory cytokines along with bronchial bile acids, have been associated with lower levels of surfactant proteins A and D [44, 45]. Although the pathophysiology remains unclear, activation of these immunomodulating proteins is consistent with the fibrotic nature of BOS, providing a theory for the detrimental effects of GORD in lung transplant patients. Moreover, Nissen fundoplication has also been associated with a decreased bacterial load, which has correlated with fewer inflammatory cytokines [46]. Reduced levels of these pro-inflammatory mediators may lead to the reduction in the rate of fibrosis and therefore, freedom from BOS in lung transplant patients providing a physiological hypothesis into the benefits of ARS on lung allograft longevity.

**Table 2:** Data regarding studies reporting on inflammatory protein in changes in lung transplant patients before and after ARS

	No. of patients		ARS	BALF pre-ARS	BALF post-ARS	Inflammatory marker decrease after ARS	Inflammatory marker increase after ARS	Inflammatory markers with no difference after ARS
Neujahr et al. 2010	8	Unspecified		20 (1–70) days	33 (14–73) days	CD8 lymphocyte, granzyme Bhi CD8	granzyme Blo CD8, CD127lo CD8, PD1hi CD8	–
Fisichella et al. 2012	8	Unspecified		4 weeks	4 weeks, 12 months	Pepsin, Lymphocytes, Neutrophils, IL-1β, IL-8	Macrophages, IFN-γ	IL-1RA, IL-2, IL-4, IL-5, IL-6, IL-7,IL-9, IL-10, IL-12, IL-13, IL-15, IL-17, G-CSF, GM-CSF, TGF-β, TNF-α, Eotaxin, IP-10, MCP-1, MIP-1α, MIP-1β, RANTES
Zhang et al. 2020	18	Nissen fundoplication		3 months	3 months	TCA, IL-1α, IL-1β, IL-8, IL-12p70, CCL5, and S100A8	–	–

There were two studies which investigated the use of ARS in lung transplant patients within the paediatric population, neither of which found convincing evidence for lung allograft outcomes [15, 29]. The reasons hypothesised for this were that there was selection bias with patients suffering from the worst reflux being referred for surgery, intra-performer variability in spirometry due to young age and associated non-compliance to instructions, and that GORD does not play a significant role in allograft dysfunction within the paediatric population.

Comparison of the surgical technique of fundoplication in this review centred around the comparison of Nissen and Toupet fundoplications. The key difference between the two techniques is that the Nissen fundoplication provides a 360-degree wrap, whilst the Toupet fundoplication creates a partial 270-degree wrap. In the current study, patients who underwent a Nissen fundoplication yielded a 2.297 mL/d slower rate of decline in FEV1 compared to those who underwent a Toupet fundoplication.

Although previous meta-analyses have shown that the two techniques are associated with similar rates of post-operative satisfaction and recurrence of GORD [47, 48], it is thought that a complete wrap creates more robust barrier against reflux by increasing pressure at the lower oesophageal sphincter to prevent reflux. Conversely, due to the full wrap involved in Nissen fundoplication rates of dysphagia, gas-bloat syndrome, inability to belch and re-operation due to severe dysphagia have previously been demonstrated to be higher than in Nissen than in Toupet fundoplications [47, 48]. Various studies in this review included the use of manometry to establish a patient’s suitability for a Nissen fundoplication, since oesophageal dysmotility is often thought to be a relative contra-indication to a Nissen fundoplication given the aforementioned complications [21, 23, 33, 39]. Pre-operative consultations for prospective ARS candidates following lung transplantation should include counselling on the benefits and effectiveness of Nissen fundoplication for improving allograft function, whilst also addressing the risks of dysphagia and other complications. In conjunction with patient’s and surgeon’s preference, other investigation results including oesophageal manometry, the most appropriate surgical technique for fundoplication, should be determined.

Radiofrequency ablation to the oesophageal sphincter via the Stretta procedure was included in a study of 11 patients this review [25]. It had the worst FEV1 and %FEV1 outcomes of all papers included in this meta-analysis with a decrease of 0.3 L/s in FEV1 and 13% in %FEV1, respectively. Furthermore, it failed to achieve statistical significance in GORD symptom resolution with a non-significant change in post-procedural pH studies and DeMeester scores (*p* = 0.95; *p* = 0.76, respectively). Seven out of 10 patients (70%) in the study ultimately went on to have a laparoscopic Toupet fundoplication. In contrast, a meta-analysis analysing the Stretta procedure out with the lung transplant setting, which included 1441 patients has demonstrated a significant improvement in health-related quality of life and average DeMeester score [49], so it is possible that there is not sufficient data to make a conclusion on this basis.

Another study of 17 patients that investigated magnetic sphincter augmentation via the LINX device was included in this review. Compared to traditional fundoplication, hospital lengths of stay were shorter although side effects including dysphagia, vomiting, residual reflux, and vomiting were more common. There were comparable rates of mortality, acute rejection, and re-intervention after a one-year follow-up [22]. Again, the limited patient pool means that these conclusions must be interpreted with caution.

Strengths of this study include the large pool of articles included in the original search and the use of various data to make conclusions. It is the first pooled analysis of studies describing survival rates and the second to describe lung function after ARS in the lung transplant population. Furthermore, investigations into publication bias through Egger’s test revealed no publication bias (Supplementary Fig. A–G).

Weaknesses include the large heterogeneity of the papers included, there were many papers with patients of various age demographics, diagnosis of GORD through different criteria, and undergoing different procedures at different timepoints following lung transplant. The studies included themselves were largely retrospective studies with no randomised trials. We were unable to do a pooled analysis on inflammatory or microbiome data due to the paucity of papers on this topic. This paper is inherently prone to selection bias since lung transplant patients who undergo ARS would likely have better allograft function and be better medically optimised than those with failing allografts.

## Conclusion

Based on this pooled analysis, ARS improves FEV1 and slows the rate of allograft decline in lung transplant recipients, potentially preventing CLAD and improving allograft survival. Fundoplication consistently reduced GORD symptoms, inflammatory markers, and microbial density in BALF, contributing to better long-term outcomes. However, data in paediatric populations and on alternative treatments like Stretta and LINX were less conclusive, with mixed outcomes and a higher incidence of adverse outcomes.

## Electronic supplementary material

Below is the link to the electronic supplementary material.

Supplementary file1 (DOCX 468 KB)

## Appendix

See Tables 3, 4, 5

**Table 3:** Search strategy using MEDLINE

1	exp Esophagus/
2	(esophag$ or oesophag$).mp
3	exp gastroesophageal reflux/
4	(gastroesophageal adj3 reflux).mp
5	(gastro adj3 oesophageal adj3 reflux).mp
6	(gastro adj3 esophageal adj3 reflux).mp
7	(gerd or gord).mp
8	exp Duodenogastric Reflux/
9	bile reflux.mp
10	(acid adj3 reflux).mp
11	(gastric adj3 acid adj3 secret$).mp
12	(stomach adj3 acid adj3 secret$).mp
13	(gastric adj3 eros$).mp
14	(stomach adj3 eros$).mp
15	Heartburn/
16	(heartburn or indigestion).mp
17	exp Esophagitis/
18	(esophagitis or oesophagitis).mp
19	(low$ adj6 sphincter$ adj3 pressur$).mp
20	Gastric Emptying/
21	Gastroparesis/
22	exp Gastritis/
23	(gastr$ adj3 empt$ adj3 disorder$).mp
24	(stomach adj3 empt$ adj3 disorder$).mp
25	Dyspepsia/
26	dyspep$.mp
27	Eructation/
28	eructation.mp
29	regurgitat$.mp
30	Hernia, Hiatal/
31	hernia$ hiat$.mp
32	1 or 2 or 3 or 4 or 5 or 6 or 7 or 8 or 9 or 10 or 11 or 12 or 13 or 14 or 15 or 16 or 17 or 18 or 19 or 20 or 21 or 22 or 23 or 24 or 25 or 26 or 27 or 28 or 29 or 30 or 31
33	Fundoplication/
34	fundoplication$.mp
35	(nissen or rossetti).mp
36	(toupet or lind or watson or besley).mp
37	(LINX or sphincter augmentation).mp
38	(STRETTA or radiofrequency ablation).mp
39	33 or 34 or 35 or 36 or 37 or 38
40	Lung Transplantation/
41	lung transplant$.tw
42	Organ Transplantation/
43	Transplantation/
44	40 or 41 or 42 or 43
45	32 and 39 and 44

**Table 4:** Search strategy using Embase database

1	exp esophagus/
2	(esophag$ or oesophag$).mp
3	exp gastroesophageal reflux/
4	(gastroesophageal adj3 reflux).mp
5	(gastro adj3 oesophageal adj3 reflux).mp
6	(gastro adj3 esophageal adj3 reflux).mp
7	(gerd or gord).mp
8	exp duodenogastric reflux/
9	bile reflux.mp
10	(acid adj3 reflux).mp
11	(gastric adj3 acid adj3 secret$).mp
12	(stomach adj3 acid adj3 secret$).mp
13	(stomach adj3 acid adj3 secret$).mp
14	(stomach adj3 eros$).mp
15	heartburn/
16	(heartburn or indigestion).mp
17	exp esophagitis/
18	(esophagitis or oesophagitis).mp
19	(low$ adj6 sphincter$ adj3 pressur$).mp
20	stomach emptying/
21	stomach paresis/
22	exp gastritis/
23	(gastr$ adj3 empt$ adj3 disorder$).mp
24	(stomach adj3 empt$ adj3 disorder$).mp
25	dyspepsia/
26	dyspep$.mp
27	eructation/
28	eructation.mp
29	eructation.mp
30	hiatus hernia/
31	hernia$ hiat$.mp
32	1 or 2 or 3 or 4 or 5 or 6 or 7 or 8 or 9 or 10 or 11 or 12 or 13 or 14 or 15 or 16 or 17 or 18 or 19 or 20 or 21 or 22 or 23 or 24 or 25 or 26 or 27 or 28 or 29 or 30 or 31
33	lung transplantation/
34	lung transplant$.tw
35	33 or 34
36	stomach fundoplication/
37	fundoplication$.mp
38	(nissen or rossetti).mp
39	(toupet or lind or watson or besley).mp
40	(LINX or sphincter augmentation).mp
41	(STRETTA or radiofrequency ablation).mp
42	36 or 37 or 38 or 39 or 40 or 41
43	32 and 35 and 42

**Table 5:** Search strategy using Cochrane Library database

ID	Search
#1	MeSH descriptor: [Esophagus] explode all trees
#2	(esophag* or oesophag*)
#3	MeSH descriptor: [Gastroesophageal Reflux] explode all trees
#4	gastroesophageal reflux
#5	gastro oesophageal reflux
#6	gord
#7	gerd
#8	MeSH descriptor: [Duodenogastric Reflux] explode all trees
#9	bile reflux
#10	acid reflux
#11	gastric acid secret*
#12	stomach acid secret*
#13	gastric eros*
#14	stomach eros*
#15	MeSH descriptor: [Heartburn] explode all trees
#16	heartburn
#17	indigestion
#18	MeSH descriptor: [Esophagitis] explode all trees
#19	esophagitis
#20	oesophagitis
#21	low* sphincter* pressur*
#22	MeSH descriptor: [Gastric Emptying] explode all trees
#23	MeSH descriptor: [Gastroparesis] explode all trees
#24	MeSH descriptor: [Gastritis] explode all trees
#25	gastr* empt* disorder*
#26	stomach empt* disorder*
#27	MeSH descriptor: [Dyspepsia] explode all trees
#28	MeSH descriptor: [Eructation] explode all trees
#29	MeSH descriptor: [Hernia, Hiatal] explode all trees
#30	dyspep*
#31	eructation
#32	regurgitat*
#33	#1 OR #2 OR #3 OR #4 OR #5 OR #6 OR #7 OR #8 OR #9 OR #10 OR #11 OR #12 OR #13 OR #14 OR #15 OR #16 OR #17 OR #18 OR #19 OR #20 OR #21 OR #22 OR #23 OR #24 OR #25 OR #26 OR #27 OR #28 OR #29 OR #30 OR #31 OR #32
#34	fundoplication*
#35	nissen or rossetti
#36	toupet or lind or watson or besley
#37	LINX or sphincter augmentation
#38	STRETTA or radiofrequency ablation
#39	MeSH descriptor: [Fundoplication] explode all trees
#40	#35 OR #36 OR #37 OR #38 OR #39
#41	MeSH descriptor: [Lung Transplantation] explode all trees
#42	lung transplant*:ti,ab,kw
#43	#41 OR #42
#44	#33 AND #40 AND #43
