# Supplementary material for: Interventional anti-reflux management for gastro-oesophageal reflux disease in lung transplant recipients: a systematic review and meta-analysis
Source: Surg Endosc. 2024 Nov 25;39(1):19–38. doi: 10.1007/s00464-024-11392-8 (PMC11666770; doi:10.1007/s00464-024-11392-8)
Supplement: Supplementary file 1 — Supplementary file1 (DOCX 468 KB) [file 464_2024_11392_MOESM1_ESM.docx]

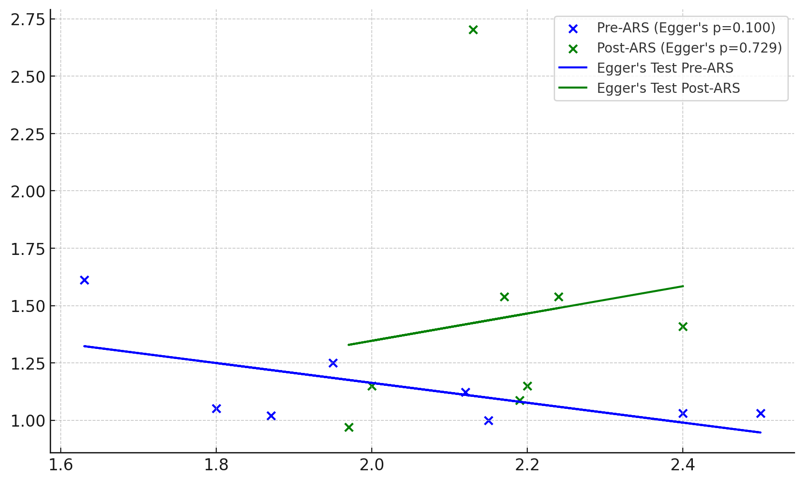


Supplementary Fig A. Egger’s test investigating risk of publication bias in studies reporting changes in FEV1 values. X-axis represents precision of study, Y-axis represents the standard normal deviate.


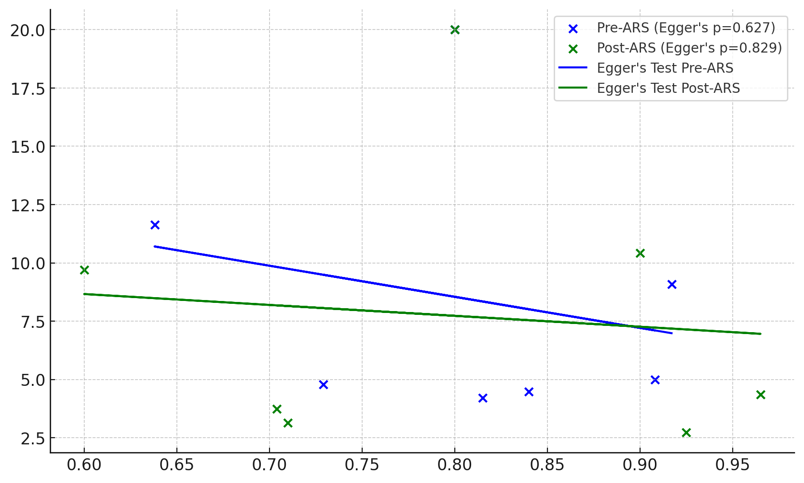


Supplementary Fig B. Egger’s test investigating risk of publication bias in studies reporting changes in % FEV1 values. X-axis represents precision of study, Y-axis represents the standard normal deviate.


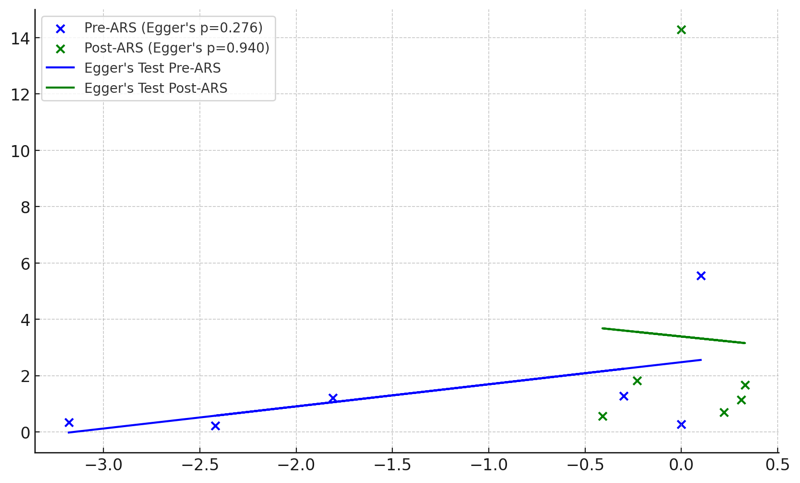


Supplementary Fig C. Egger’s test investigating risk of publication bias in studies reporting changes in rate of change of FEV1. X-axis represents precision of study, Y-axis represents the standard normal deviate.


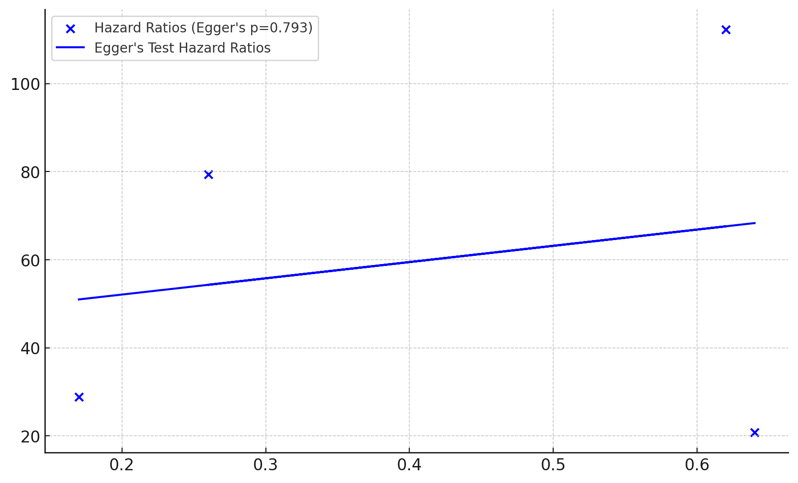


Supplementary Fig D. Egger’s test investigating risk of publication bias in studies reporting univariate survival data. X-axis represents precision of study, Y-axis represents the standard normal deviate.


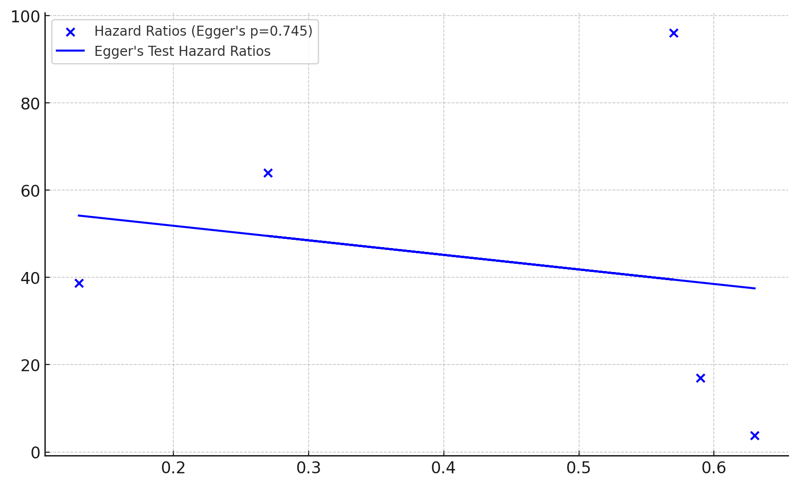


Supplementary Fig E. Egger’s test investigating risk of publication bias in studies reporting multivariate survival data. X-axis represents precision of study, Y-axis represents the standard normal deviate.


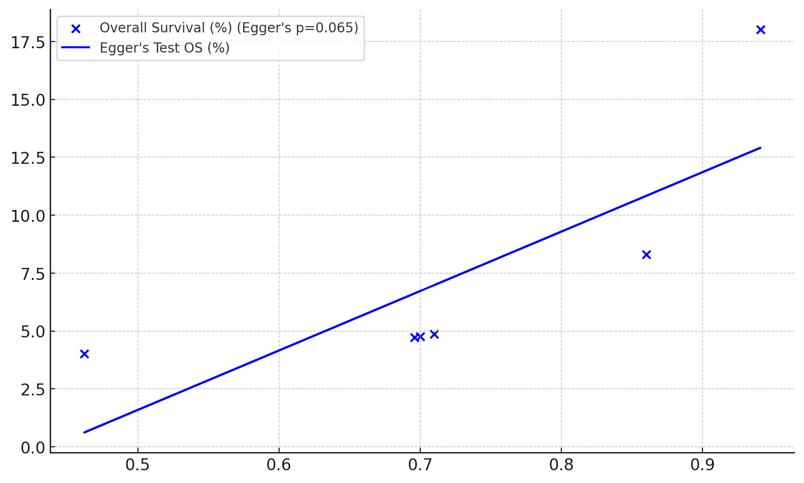


Supplementary Fig F. Egger’s test investigating risk of publication bias in studies reporting overall survival data in those who underwent ARS. X-axis represents precision of study, Y-axis represents the standard normal deviate.


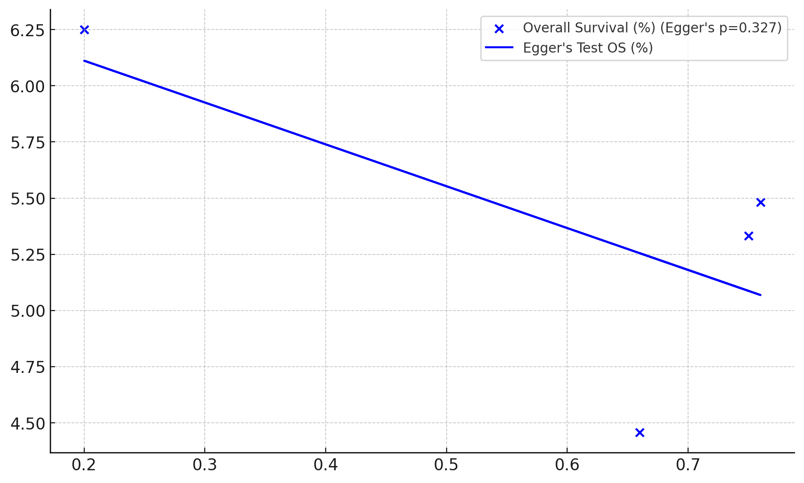


Supplementary Fig G. Egger’s test investigating risk of publication bias in studies reporting overall survival data in lung transplant patients who did not undergo ARS. X-axis represents precision of study, Y-axis represents the standard normal deviate.
